# TCR and CD28 activate the transcription factor NF-κB in T-cells via distinct adaptor signaling complexes

**DOI:** 10.1016/j.imlet.2014.10.020

**Published:** 2015-01

**Authors:** Youg Raj Thaker, Helga Schneider, Christopher E. Rudd

**Affiliations:** Cell Signalling Section, Department of Pathology, University of Cambridge, Tennis Court Road, Cambridge CB2 1QP, United Kingdom

**Keywords:** CD, cluster of differentiation (e.g. CD28), TCR, T-cell receptor (TCRαβ), NF-κB, nuclear factor κB, IκB, inhibitor of NF-κB, PBL, peripheral blood lymphocytes, SKAP1, Src-kinase associated phosphoprotein 1, Adaptors, ADAP, VAV-1, GRB-2, NF-κB

## Abstract

The transcription factor NF-κB is needed for the induction of inflammatory responses in T-cells. Whether its activation by the antigen-receptor and CD28 is mediated by the same or different intracellular signaling pathways has been unclear. Here, using T-cells from various knock-out (*Cd28*^−/−^, *adap*^−/−^) and knock-in (i.e. *Cd28* Y-170F) mice in conjunction with transfected Jurkat T-cells, we show that the TCR and CD28 use distinct pathways to activate NF-κB in T-cells. Anti-CD28 ligation alone activated NF-κB in primary and Jurkat T-cells as measured by NF-κB reporter and EMSA assays. Anti-CD28 also activated NF-κB normally in primary T-cells from *adap*^−/−^ mice, while anti-CD3 stimulation required the adaptor ADAP. Over-expression of ADAP or its binding partner SKAP1 failed to enhance anti-CD28 activation of NF-κB, while ADAP greatly increased anti-CD3 induced NF-κB activity. By contrast, CD28 activation of NF-κB depended on GRB-2 binding to CD28 as seen in CD28 deficient Jurkat T-cells reconstituted with the CD28 YMN-FM mutant, and in primary T-cells from CD28 Y170F mutant knock-in mice. CD28 associated with GRB-2, and GRB-2 siRNA impaired CD28 NF-κB activation. GRB-2 binding partner and guanine nucleotide exchange factor, VAV1, greatly enhanced anti-CD28 driven activation of NF-κB. Further, unlike in the case of anti-CD28, NF-κB activation by anti-CD3 and its cooperation with ADAP was strictly dependent on LAT expression. Overall, we provide evidence that CD28 and the TCR complex regulate NF-κB via different signaling modules of GRB-2/VAV1 and LAT/ADAP pathways respectively.

## Highlights

• CD28 and TCR receptors use independent pathways to regulate NF-κB activation in T-cells.
• CD28 mediated NF-κB activation is dependent on the YMN-FM site for GRB-2 adaptor binding.
• The adaptors ADAP and SKAP1 are dispensable for direct CD28 activation of NF-κB.
• TCR driven NF-κB activation requires adaptor ADAP expression.

## 1 Introduction

T-cell activation is controlled by the engagement of the T-cell receptor (TCR) and an array of co-stimulatory and co-inhibitory molecules that encounter binding partners during antigen presentation [1–3]. CD28 is a co-receptor that is engaged by ligands CD80 and CD86 on antigen presenting cells (APCs) [4,5]. The activation process is accompanied by the clustering of CD28 in a manner that regulates the localization of protein kinase C theta, a marker for the formation of central supramolecular activation complex (cSMAC) [6–8]. CD28 is required for productive T-cell responses and survival [5,9–12,60]. Engagement of TCR without co-stimulation can lead to anergy, a state of hypo-responsiveness [13,14]. Further, CD28 regulates metabolism and the mRNA stability of multiple cytokines including IL-2 [15]. CD28 deficient mice show defects in proliferation, cytokine production and cell survival [16,17]. Mutational analysis of IL-2 promoter has identified the NF-κB binding site on CD28 response element (CD28RE) [18] as well as other sites that are responsive to stimulation by the TCR [19,20]. In this context, the transcription factor NF-κB is regulated by ligation of both CD3 and CD28 and is essential for T-cell activation [21,22].

T-cell activation is mediated by an array of protein kinases and adaptor proteins, the latter integrating signals for activation [1,25]. Adaptors lack enzymatic activity and transcription binding domains, but facilitate protein-protein interactions [26]. The family of adaptors in T-cells includes linker for activation of T-cells (LAT), lymphocyte cytosolic protein-2 (SLP-76), adaptor adhesion and degranulation promoting adaptor protein (ADAP or Fyb), VAV1 and SKAP1 [1,27,28]. Recently, role of adaptors have emerged in the regulation of NF-κB. ADAP can be recruited to the Carma1-Bcl10-Malt1 (CBM) complex upon CD3/CD28 stimulation, a key complex that regulates NF-κB activation via the classical pathway [29,30]. Also, a role for the multi-domain adaptor VAV1 has been noted in the CD28-NF-κB pathway [31]. VAV1 is a GTP exchange factor (GEF) protein for Rho/Rac family of GTPases required for the optimal effector responses and cytoskeleton actin polymerization [32,33]. Unlike in the case of TCR and CD28, Toll receptors and IL-1 of innate signaling use the MyD88 adaptor-like, TRAF-6, and Rac1 for the activation of NF-κB [23,24].

Despite the importance of CD28 in potentiating TCR activation of T-cells (i.e. co-stimulation), increasing evidence has shown that its ligation alone can induce signaling events in T-cells [31,34,35]. For example, mitogenic anti-CD28 can induce proliferation in absence of TCR ligation [36]. Further, non-mitogenic anti-CD28 can regulate NFAT transcription via VAV1/SLP-76 adaptors independently of TCR ligation [34]. We previously showed that GEF VAV1 binding to CD28 involves the intermediate binding of another adaptor GRB-2 [37,38]. GRB-2 is a classic adaptor comprised of an SH2 domain flanked by an N- and C-terminal SH3 domain. Abe and coworkers have previously shown that the loss of either GRB-2 or GADS binding to CD28 can abrogate NK-κB activation in Jurkat T-cells [39]. In addition, Tuosto et al. have reported that non-mitogenic anti-CD28 can deliver a unique signal leading to the recruitment of p52/Rel-A complexes on Bcl-xL promoter [40]. They showed that CD28 can co-operate with VAV-1 to activate NF-κB in a pathway involving Rac-1 and mitogen-activated kinase kinase 1 [31,41].

Despite this progress, studies dissecting the individual components of TCR and CD28-mediated NF-κB activation in primary T-cells have been lacking. In this paper, using primary T-cells from various knock-out (*Cd28*^−/−^, *adap*^−/−^) and knock-in (i.e. *Cd28* Y-170F) mice in conjunction with transfected Jurkat T-cells, we show that the TCR and CD28 use distinct pathways for the activation of the NF-κB pathway in T-cells. Our findings provide further evidence that the CD28 and TCR pathways regulate NF-κB activity via different signaling modules of GRB-2/VAV1 and LAT/ADAP respectively.

## 2 Materials and methods

### 2.1 Mice and isolation of T-cells

DO11.10-CD28 KO and CD28 Y170F knock-in mutant mice (kindly provided by Dr. Jonathan Green, Washington University School of Medicine); C57BL/6-ADAP KO mice (kindly provided by Dr. Erik Peterson, University of Minnesota, MN) were bred and housed under pathogen free conditions at the Central Biomedical facility (CBS), University of Cambridge; Gurdon Institute, Animal Facility Unit, University of Cambridge; or Department of Pathology, Animal Unit (BSU), University of Cambridge. CD3^+^ cells were enriched from splenocytes using a negative selection column kit (R&D Systems). Purity of isolated T-cells was greater than 90%.

### 2.2 Cell culture and antibodies for flow cytometry and activation

Mouse T-cells were cultured in RPMI 1640 supplemented with 10% fetal bovine serum (FBS, Sigma), 2 mM l-glutamine, 100 U/ml penicillin/streptomycin and 50 uM β-mercapto-ethanol at 37 degrees in 5% humidified chamber. Jurkat T-cells were maintained in 5–10% FBS and 2 mM l-glutamine. Human anti-CD3 (OKT3) was obtained from American Type Culture Collection, human anti-CD28 (CD28.2 clone, BD Pharmingen), FITC labeled anti-human CD28 (Cat. no. 556621, BD Pharmingen), anti-mouse CD3 antibody (2C11 clone, Bioexpress) and mouse anti-CD28 antibody (PV-1 clone, Bioexpress).

### 2.3 IL-2 NF-κB minimal promoter activity

T-cells were transfected with IL-2 promoter binding sites NF-κB luciferase (firefly) reporter plasmid together with Renilla luciferase plasmid (pRLTK, Promega) as an internal control to adjust for the transfection efficiency and background. Whenever described in Results section cells were co-transfected with other effector plasmids in conjunction with empty vectors to adjust total amount of DNA. Following 24 h of expression, murine T-cells were treated with anti-CD28 (PV1) or anti-CD3 (2C11) for 6 h. Jurkat T-cells were stimulated with anti-CD28 (CD28.2) or anti-CD3 (OKT3) antibodies and lysed in 100 μl of passive lysis buffer provided with dual luciferase assay kit (Promega). Light units were recorded on Luminometer (Berthold) from 10 μl of sample in 50 μl substrate solution as per the manufacturer's instructions. Relative luciferase units were derived by normalizing values relative to the Renilla values. Each sample was measured in triplicates and final average values were plotted with standard deviations. Each experiment was repeated at least three times.

### 2.4 Transfections of Jurkat and primary cells, immunoprecipitation and blotting

Primary T-cells were transfected with 4 μg of DNA per 8 million cells using mouse or human Nucleofactor kit (Lonza). Briefly, cells were washed two times with PBS and resuspended in a mixture of solution A and B (4.5:1 ratio) plus plasmid(s) and pulsed using optimized protocol for CD4^+^ cells or human PBLs on Nucleofactor 2b device. Jurkat T-cells were transfected with 1–2 μg of DNA per 1 million cells in RPMI without FBS and pulsed with a unipolar pulse (310 V, 10 ms) on BTX electroporator. Cells were immediately transferred to pre-equilibrated RPMI-1640 containing 10% FBS and l-glutamine without antibiotics. Cells were lysed in NP-40 lysis buffer supplemented with protease inhibitor cocktail (Roche), immunoprecipitated with 2 μg of antibodies for 2 h at 4 degrees. Immuno complexes were captured by protein G beads (GE Healthcare) and washed 4 times with lysis buffer and heated in loading buffer. All samples were loaded onto 10% SDS gel (Novex, Invitrogen) and transferred onto PVDF membrane, followed by blotting with primary and respective secondary antibodies.

### 2.5 Electromobility shift assay

CD3^+^ T-cells were stimulated with control or anti-CD28 antibodies for 6 h at 37 degrees. Cells were harvested, lysed in hypotonic buffer and nuclear fractions were isolated using nuclear extract kit (ActiveMotif) as per the manufacturer's instructions. Protein concentration was quantified using BCA protein assay (Pierce). 4 μg of protein were used in each condition. A non-radioactive NF-κB electromobility shift assay (EMSA) was performed as per the manufacturer's instructions (Panomics, Affimatrix) using biotinylated NF-κB probes and provided positive and negative controls.

### 2.6 siRNA knock-down

Control and gene-specific siRNA were purchased from Dharmacon (Thermo Scientific) and transfected into either primary or Jurkat T-cells as described above. Cells were harvested for analysis after 36 h or longer as described in the specific experiment.

## 3 Results

### 3.1 CD28 and TCR regulate and synergize the NF-κB activation in T-cells using independent and unique pathways

**Fig. 1:**
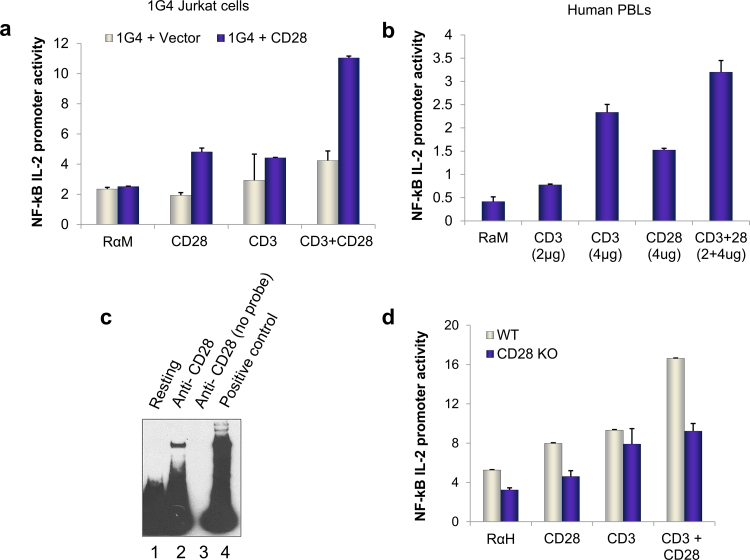
CD28 and TCR uniquely regulated NF-κB. (a) 1G4 CD28 deficient Jurkat cells reconstituted with either empty plasmid (vector) or WT (CD28) untagged plasmid were used to measure NF-κB IL-2 promoter luciferase (firefly) activity in response to indicated ligating antibodies. Activity was measured by normalizing firefly luciferase counts to the background Renilla luciferase values, done in triplicates for each point as described in Materials and Methods section. All values are in relative luciferase units. (b) NF-κB activation in CD3^+^ human peripheral blood lymphocytes in response to engagement with indicated concentrations of CD3 (OKT3 clone) or CD28 (CD28.2 clone) alone or in combination. (c) Electromobility shift assay from nuclear fractions of C57BL/6 naïve T-cells as described in section 2. Briefly, a biotinylated NF-κB probe was used to detect DNA binding to nuclear fraction from unstimulated (lane 1) or anti-CD28 stimulated cells (lane 2). For specificity, anti-CD28 stimulated nuclear fraction was probed with unlabeled/cold NF-κB oligo (lane 3). Lane 4 is positive control (provided with kit). (d) NF-kB activation in primary T-cells isolated from WT or CD28 KO mice treated with control (RαH), anti-CD3 or anti-CD28 alone stimulating antibodies or in combination. Data in each sample is average of triplicates. All antibodies concentrations are in micrograms per milliliter of solution.

CD28 and TCR regulation of NF-κB activation is well established [40,42,43]. Whether CD28 and TCR use different pathways to regulate NF-κB is not known. To assess this issue, anti-CD28 was initially used in conjunction with anti-CD3 to examine effects on NF-κB activation. A reporter construct carrying the NF-κB binding sites of the interleukin 2 promoter was transfected into cells and used as a readout [44,45]. Initially, 1G4 Jurkat T-cells deficient for CD28 expression were transfected with CD28 and ligated with a non-mitogenic anti-CD28 and/or anti-CD3 antibodies (Fig. 1a). Anti-CD28 alone induced an increase in NF-κB transcription on the IL-2 gene as reported [31,40]. Co-ligation of CD28 with CD3 co-operated to produce higher transcription of luciferase gene (Fig. 1a). Similar findings were observed in human peripheral T-cells transfected with the NF-κB reporter construct (Fig. 1b). Anti-CD28 alone induced an increase in NF-κB promoter and cooperated with anti-CD3 ligation to further enhance NF-κB activity. An electromobility shift assay (EMSA) from nuclear extracts of anti-CD28 stimulated primary cells were probed with biotin-labeled NF-κB probe and showed significant NF-κB DNA binding (Fig. 1c). As a control, T-cells from *Cd28*^*−*/*−*^ mice showed a lack of NF-κB activation by anti-CD28. Despite this, anti-CD3 retained an ability to stimulate NF-κB reporter activity (Fig. 1d). However, both anti-CD3 and CD28 driven pathways were normal in control animals. These data indicated that anti-CD3 can activate NF-κB transcription independently of the expression of the CD28 on T-cells.

### 3.2 CD28 requires GRB-2 binding to regulate NF-κB activation independent of TCR ligation

**Fig. 2:**
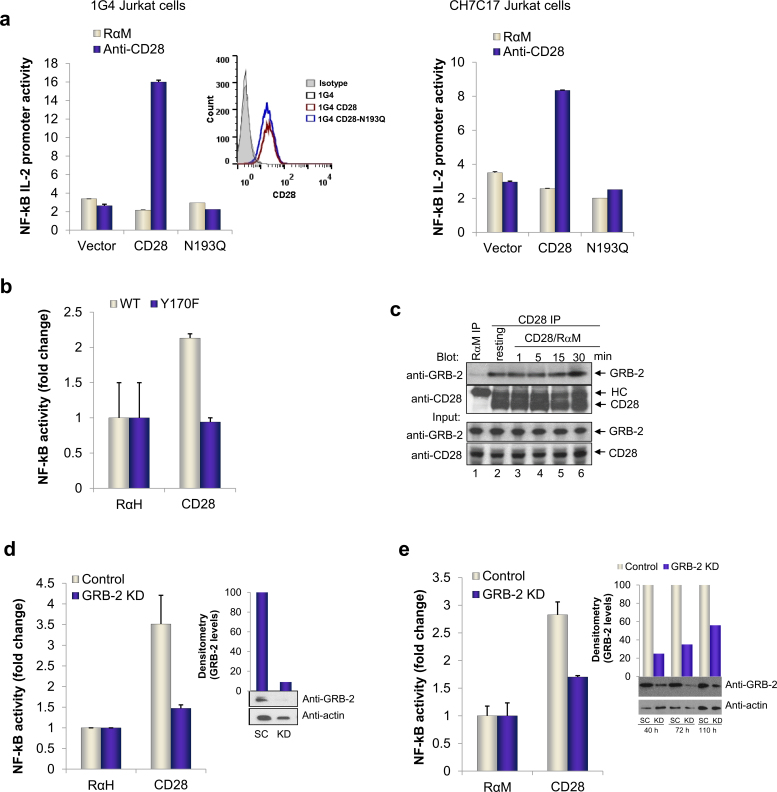
GRB-2 connects proximal signals to regulate CD28 driven NF-κB pathway. (a) *Cd28*^−/−^ cells were reconstituted with empty vector, WT CD28 or N193Q mutant. FACS histogram shows surface expression of WT or N193Q CD28 by staining with FITC labeled antibodies to CD28. Right panel shows another representative experiment obtained from CH7C17, CD28 deficient cell line. NF-κB luciferase activity was measured after stimulation with indicated antibodies. (b) Y170F knock-in mutant primary T-cells were used in conjunction with wild type primary cells. Luciferase firefly units were normalized to background Renilla values. (c) Binding of Grb-2 to CD28. Jurkat T-cells, either left unstimulated (lane 2) or stimulated with anti-CD28/rabbit anti-mouse IgG for 1, 5, 15 and 30 min (lanes 3–6) were lysed, immunoprecipitated with anti-CD28 and blotted for Grb-2. Immunoprecipitation with rabbit anti-mouse IgG served as a negative control (lane 1). Input panels: blotting of cell lysates with Grb-2 (upper panel) or CD28 (lower panel) served as a loading control. siRNA knock-down of GRB-2 in primary cells (d) and Jurkat cells (e) and its effects on NF-κB pathway after 48 and 72 h of knock-down respectively. Efficiency of knock-down was assessed by western blotting as shown in insets (SC: scrambled and KD: knock-down) and quantified by normalizing to endogenous actin levels (inset column chart). All siRNA were purchased from Dharmacon (Thermo Scientific).

To dissect the nature of the CD28 proximal signaling pathway, we initially made use of Jurkat cell lines, 1G4 and CH7C17, deficient for CD28 expression that were reconstituted by transfection with either WT CD28 (WT), or a mutant form of CD28 (N193Q) (Fig. 2a). Cell clones stably expressing CD28 and N193 were selected and used for all subsequent experiments. FACS analysis showed equal levels of CD28 and N193Q surface expression on the cells (inset). We had previously shown that the N193Q mutation selectively interferes with GRB-2 binding, without affecting the binding of PI3K [42,46]. While anti-CD28 induced an increase reporter activity in the CD28 reconstituted cells (i.e. 6- to 7-fold increase) (left hand panel), cells expressing the N193Q mutant showed an almost complete impairment of NF-κB activation, identical to the unstimulated control. Similar results were obtained with CH7C17 Jurkat cell line (right hand panel). Further, we showed that primary cells from CD28 knock-in mutant (Y170F), which disrupts binding of PI-3 kinase and GRB-2 to its YMNM motif also showed a complete loss of NF-κB activation by anti-CD28 (Fig. 2b).

**Supplementary Figure S1:**
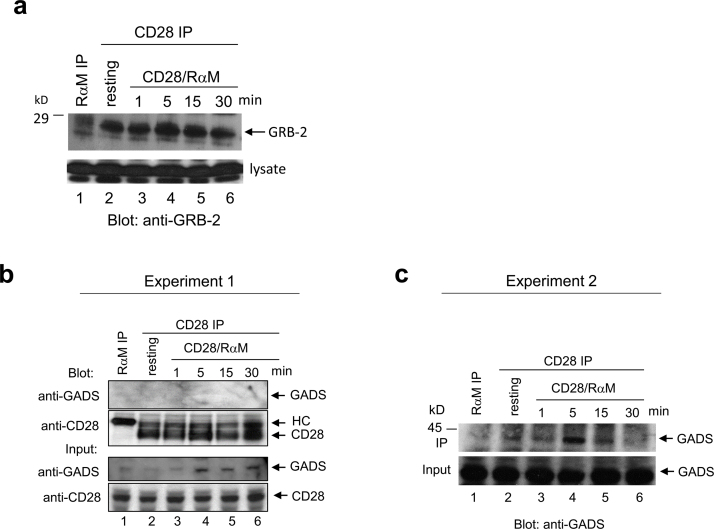
Binding of Grb-2 and GADS to CD28. Jurkat T-cells, either left unstimulated (lane 2) or stimulated with anti-CD28/rabbit anti-mouse IgG for 1, 5, 15 and 30 min (lanes 3-6) were lysed, immunoprecipitated with anti-CD28 and blotted for Grb-2 (a) or GADS (b, c). Immunoprecipitation with rabbit anti-mouse IgG served as a negative control (lane 1). Input panels: blotting of cell lysates with Grb-2 or Gads served as a loading control.

A previous report had attributed this effect to a loss of either GRB2 or GADS binding to CD28 [39]. To assess the relative binding of each to CD28 in Jurkat cells, the co-receptor was ligated followed by precipitation and blotting with either anti-GADS (Supp. Fig. 1b and c) or anti-GRB2 (Fig. 2c). In agreement with our previous reports [37,46], GRB-2 was co-precipitated at substantive levels over the time course of 1–30 min by anti-CD28 (lanes 2–6). Binding was also observed in resting cells (lane 2), and occasionally anti-CD28 ligation resulted in an increased association (Supp. Fig. 1a, lane 4). Blotting of cell lysates showed similar amounts of GRB-2 and CD28 (lower panel). By contrast, while similar amounts of GADS and CD28 could be detected in the cell lysates (Supp. Fig. 1b and c), anti-CD28 co-precipitated little or no GADS as compared to GRB-2 (Supp. Fig. 1b and c vs. Fig. 2c). In many experiments, GADS could not be detected with anti-CD28 precipitates. This observation suggested that GRB-2 was the adaptor primarily responsible for CD28 proximal complex assembly.

To establish this in primary and Jurkat T-cells, siRNA was used to down-regulate GRB-2 expression followed by an assessment of anti-CD28 induction of NF-κB reporter activation. From this, it was clear that the knock-down of GRB-2 in primary and Jurkat T-cells blocked anti-CD28 mediated NF-κB activation (Fig. 2d and e). The efficiency of knock-down was confirmed by western blotting of total protein levels (insets) and quantified relative to endogenous actin levels by densitometric analysis. The use of primary and Jurkat T-cells showed that anti-CD28 activation of NF-κB required GRB-2 expression and its binding to CD28.

### 3.3 The adaptor ADAP is not needed for direct CD28 activation of NF-κB

**Fig. 3:**
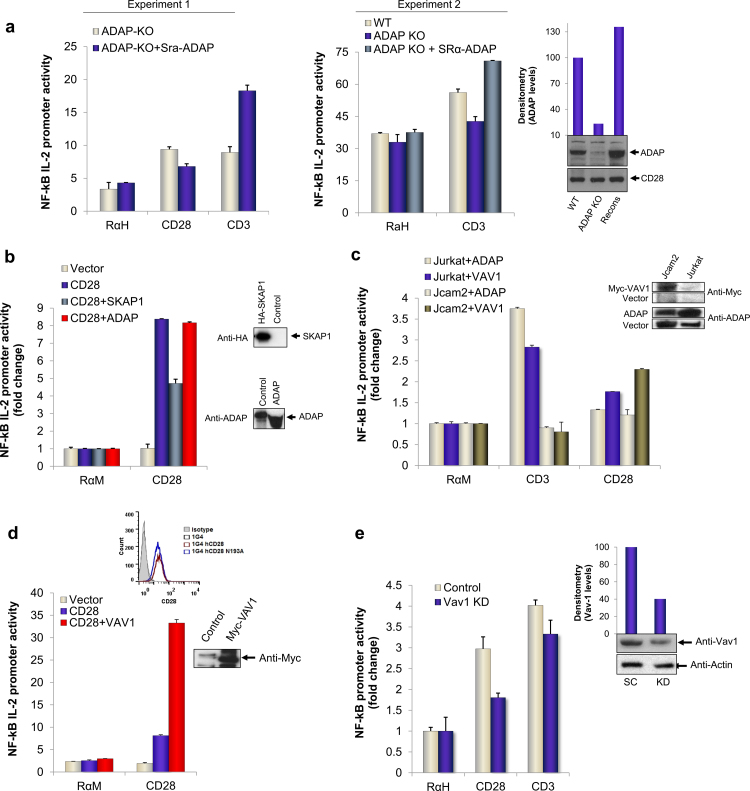
Normal CD28 but defective TCR mediated NF-κB pathway in ADAP KO primary and LAT deficient Jurkat cells*.* (a) CD3^+^ cells isolated from spleens of *adap*^−/−^ (silver bars) or wild type (blue bars) mice were used for NF-κB IL-2 promoter assay by transfecting with reporter plasmids followed by stimulation with control, anti-CD28 or anti-CD3 antibodies. Right panel, NF-κB activity in *adap*^−/−^ (blue bars) compared to controls wild type (silver bars) or *adap*^−/−^ T-cells transfected with exogenous ADAP (green bars). Efficiency of transfection was assessed by western blotting and normalized to endogenous CD28 levels as shown by column chart (inset). Each point is average of triplicate with SD. (b) NF-κB activity measured in CD28 reconstituted Jurkat 1G4 cells transfected with adapters SKAP1 or ADAP (Fyb) or control plasmids as shown. NF-κB activity was measured as above. (c) NF-κB reporter activity in LAT deficient (Jcam2) cells, either transfected with VAV1 (green bars) or ADAP (silver bars) when engaged with control (RaM), anti-CD28 or anti-CD3 antibodies. Wild type (Jurkat) transfected with respective plasmids were used as control. All values are relative luciferase units and average of triplicates with standard deviations. (d) Reconstitution of *Cd28*^−/−^ Jurkat cells with VAV1 or respective controls. NF-κB activation in response to CD28 was measured as above. (e) siRNA knock-down of VAV1 in primary T-cells for 48 h. NF-κB activity was measured from reporter plasmids when cells were stimulated for 6 h with control (RαH), anti-CD28 or anti-CD3 antibodies. Knock-down efficiency was detected by western blotting of endogenous proteins and quantified by densitometric analysis and shown as actin normalized values in column chart (inset). (For interpretation of the references to color in this figure legend, the reader is referred to the web version of this article.)

Previous reports have shown a role of ADAP-CARMA1-Bcl10-Malt1 complex in the regulation of NF-κB in T-cells in response to co-ligation by TCR/CD28 [47–49]. However, the role of ADAP in anti-CD28 stimulation alone of NF-κB had not been examined. To assess this, we next examined the effects of anti-CD28 on primary T-cells from *adap*^−/−^ mice (Fig. 3a). Anti-CD28 induced the normal 2- to 4-fold increase in reporter activity in *adap*^−/−^ T-cells. Further, transfection of the cells with ADAP failed to increase this further, and occasionally even reduced NF-κB activity. In contrast, anti-CD3 NF-κB activation was impaired in *adap*^−/−^ cells as compared to wild type cells which showed normal NF-κB activation. As a control, the reconstitution of primary cells with exogenous ADAP restored normal or higher levels of NF-κB activation in response to anti-CD3. Levels of over-expressed ADAP in reconstituted cells were comparable to endogenous ADAP levels expressed by wild type cells (inset). No substantial differences in CD28 expression were observed on *adap*^−/−^ cells as measured by total CD28 blot (inset). Similarly, anti-CD28 was used to activate NF-κB reporter activity in vector or CD28 expressing Jurkat 1G4 cells transfected with ADAP or SKAP1 (Fig. 3b). ADAP binds to SKAP1 in a pathway linked to the activation of integrin adhesion [50,51]. Again, the over-expression of neither ADAP nor SKAP1 enhanced anti-CD28 induced NF-κB activity, and occasionally, we even observed an inhibition of activity. Immune blotting confirmed expression of transfected proteins in cells (right insets).

In another approach, we also examined whether ADAP expression could potentiate anti-CD3 or anti-CD28 activation of NF-κB in LAT deficient Jcam2 T-cells (Fig. 3c). LAT is an adaptor that operates upstream of ADAP in the anti-CD3 activation cascade in a number of contexts [2,52–54]. Interestingly, anti-CD28 could readily activate NF-κB in the absence of LAT expression. By contrast, anti-CD3 was completely impaired in the activation of NF-κB reporter activity in LAT deficient cells, but not wild type cells, as was its further enhancement by the transfection with ADAP (Fig. 3a, c). This observation underscored the fundamental differences in the CD28 and TCR regulation of NF-κB, indicating that anti-CD3 mediation of NF-κB activation but not CD28 was dependent on the LAT adaptor.

Given the lack of a connection between CD28 and ADAP, we next assessed whether another adaptor may play a role in mediating CD28 effects. We previously showed that GRB-2 binding to CD28 involved the additional binding to the GEF VAV1 [37,38]. Tuosto et al. have reported that VAV1 can cooperate with CD28 to induce NF-κB activation via a pathway involving Rac-1 and mitogen-activated kinase kinase 1 [31,41]. In agreement, transfection of Jurkat cells with VAV1 resulted repeatedly in a major increase in NF-κB promoter activation (i.e. 4-fold increase) as compared to vector-transfected cells (Fig. 3d). VAV1 expression by transfection was confirmed by immune blotting (inset) and surface expression of CD28 expressing clones was analyzed by FACS (inset FACS histogram). Further, the siRNA knock-down of VAV1 expression (see inset) impaired the ability of anti-CD28 to increase NF-κB activity (Fig. 3e). As quantified, siRNA depleted about 60% of endogenous protein (inset graph). By contrast, only minor effect was seen on anti-CD3 mediated NF-κB activation. Further, we also observed that the ability of VAV1 transfection to cooperate with anti-CD28 could be seen in LAT deficient Jcam2 cells (Fig. 3c). Overall, this observation showed that CD28 co-operated with VAV1 to augment NF-κB activity and this operated independently of the LAT adaptor.

## 4 Discussion

Overall, our findings show that CD28 and the TCR complex engaged distinct signaling modules, GRB-2/VAV1 and LAT/ADAP respectively, in the activation of NF-κB. These observations could have important implications for our understanding of the intracellular signaling mechanisms that control T-cell function, and underscore the independent nature of signaling by CD28. CD28 can enhance signaling via the antigen-receptor [55], but also has the capacity to complement the TCR complex by engaging an independent pathway for the convergent activation of NF-κB. Previous studies have reported the importance of both TCR and CD28 receptors in the activation of NF-κB [56,57]. However, despite this accumulating evidence showing CD28 independent signaling in T-cells, most previous studies have examined responses mostly in Jurkat cells. The potential definition of distinct pathways in primary T-cells had been missing. Importantly, recent studies have implicated the adaptor ADAP (alias FYB, SLAP-130) in the activation of NF-κB in response to anti-CD3/CD28 co-ligation. It has been unclear whether independent CD28 signaling also requires or uses the ADAP pathway. Using adap^−/−^ primary T-cells, we found that anti-CD28 could activate NF-κB without the expression of ADAP. This contrasted with the TCR complex where NF-κB activation was markedly impaired in the absence of ADAP. Further, in a complementary approach, while the over-expression of ADAP substantially increased anti-CD3 mediated of NF-κB reporter activity, the over-expression of ADAP, or its binding partner SKAP1 (alias SKAP-55) had little if any effect in anti-CD28 driven activation. Consistent with this theme, while anti-CD3 activation of NF-κB was markedly impaired in LAT deficient Jcam2 cells, anti-CD28 readily activated NF-κB in these T-cells. This observation was in keeping with the close connection between the TCR and LAT in the activation of T-cells [2]. It is also consistent with the notion that the LAT signalosome is comprised of a complex that binds to SLP-76 and which in turn binds via its SH2 domain to ADAP [1,53,58].

Overall, while the TCR pathway depends on LAT and associated ADAP to activate the NF-κB pathway, our data show that CD28 uses a distinct pathway that does not depend on this pathway. Instead, we found that GRB-2 and VAV1 are key elements in the CD28 pathway. We have previously shown that GRB-2 can bind via two domains to CD28. Further, we have shown that GRB-2 can bind to VAV1 when associated with CD28 [38]. In particular, CD28 and GRB-2, relative to Gads or Grap, can preferentially cooperate with VAV1 in the activation of NFAT/AP-1 transcription [38]. Given the previous finding that the loss of GADS or GRB-2 was needed for CD28 activation of NF-κB in Jurkat cells [39], we reassessed the binding of GADS and GRB-2, and found that only GRB-2 reproducibly bound to CD28. Further, the loss of the N residue in the YMNM motif in Jurkat cells, or the Y in primary T-cells abrogated anti-CD28 induction of NF-κB activation. We also showed that anti-CD28 cooperated with VAV1 as reported by others [31]. Further, the knock-down of GRB-2 by siRNA markedly impaired NF-κB activation in primary and Jurkat cells in response to anti-CD28. Combined with our previous demonstration that GRB-2 can bind to VAV1 [34], these findings are consistent with a model where CD28 can employ GRB-2 to engage VAV1 in the activation of NF-κB. CD28 could use the YMNM/GRB-2 as an initiation switch to regulate VAV1/SLP-76 module toward NF-κB activation.

Overall, these findings highlighted the distinct requirements for the TCR and CD28 in the activation of NF-κB pathway. It seems a reasonable assumption that TCR and CD28 would engage distinct mediators that in turn complement each other for the full activation of NF-κB transcription in T-cells. This might also provide a mechanistic explanation for the partial defects in NF-κB activation that have been observed in CD28 and ADAP KO mice. The distinct upstream mediators may also induce distinct down-stream complexes as in previous report showing that CD28 ligation induces complexes of Bcl10-PKCθ [59] that are distinct from CBM-ADAP complex of TCR pathway [29].

## References

[1] Rudd C.E. (1999). Adaptors and molecular scaffolds in immune cell signaling. Cell.

[2] Samelson L.E. (2002). Signal transduction mediated by the T cell antigen receptor: the role of adapter proteins. Annu Rev Immunol.

[3] Weiss A. (2009). TCR signal transduction: opening the black box. J Immunol.

[4] Dustin M.L., Shaw A.S. (1999). Costimulation: building an immunological synapse. Science.

[5] June C.H., Bluestone J.A., Nadler L.M., Thompson C.B. (1994). The B7 and CD28 receptor families. Immunol Today.

[6] Freiberg B.A., Kupfer H., Maslanik W., Delli J., Kappler J., Zaller D.M. (2002). Staging and resetting T cell activation in SMACs. Nat Immunol.

[7] Yokosuka T., Kobayashi W., Sakata-Sogawa K., Takamatsu M., Hashimoto-Tane A., Dustin M.L. (2008). Spatiotemporal regulation of T cell costimulation by TCR-CD28 microclusters and protein kinase C theta translocation. Immunity.

[8] Dustin M.L., Bivona T.G., Philips M.R. (2004). Membranes as messengers in T cell adhesion signaling. Nat Immunol.

[9] Salomon B., Lenschow D.J., Rhee L., Ashourian N., Singh B., Sharpe A. (2000). B7/CD28 costimulation is essential for the homeostasis of the CD4+CD25+ immunoregulatory T cells that control autoimmune diabetes. Immunity.

[10] Alegre M., Fallarino F., Zhou P., Frauwirth K., Thistlethwaite J., Newell K. (2001). Transplantation and the CD28/CTLA4/B7 pathway. Transplant Proc.

[11] Riley J.L., June C.H. (2005). The CD28 family: a T-cell rheostat for therapeutic control of T-cell activation. Blood.

[12] Zang X., Allison J.P. (2007). The B7 family and cancer therapy: costimulation and coinhibition. Clin Cancer Res.

[13] Frauwirth K.A., Alegre M.L., Thompson C.B. (2000). Induction of T cell anergy in the absence of CTLA-4/B7 interaction. J Immunol.

[14] Frauwirth K.A., Riley J.L., Harris M.H., Parry R.V., Rathmell J.C., Plas D.R. (2002). The CD28 signaling pathway regulates glucose metabolism. Immunity.

[15] Lindstein T., June C.H., Ledbetter J.A., Stella G., Thompson C.B. (1989). Regulation of lymphokine messenger RNA stability by a surface-mediated T cell activation pathway. Science.

[16] Shahinian A., Pfeffer K., Lee K.P., Kundig T.M., Kishihara K., Wakeham A. (1993). Differential T cell costimulatory requirements in CD28-deficient mice. Science.

[17] Jenkins M.K. (1994). The ups and downs of T cell costimulation. Immunity.

[18] Shapiro V.S., Truitt K.E., Imboden J.B., Weiss A. (1997). CD28 mediates transcriptional upregulation of the interleukin-2 (IL-2) promoter through a composite element containing the CD28RE and NF-IL-2B AP-1 sites. Mol Cell Biol.

[19] Jain J., Loh C., Rao A. (1995). Transcriptional regulation of the IL-2 gene. Curr Opin Immunol.

[20] Chow C.W., Rincon M., Davis R.J. (1999). Requirement for transcription factor NFAT in interleukin-2 expression. Mol Cell Biol.

[21] Cheng J., Montecalvo A., Kane L. (2011). Regulation of NF-kB induction by TCR/CD28. Immunol Res.

[22] Hayden M.S., West A.P., Ghosh S. (2006). NF-kappaB and the immune response. Oncogene.

[23] Brikos C., O’Neill L.A. (2008). Signalling of toll-like receptors. Handb Exp Pharmacol.

[24] Jefferies C., Bowie A., Brady G., Cooke E.L., Li X., O’Neill L.A. (2001). Transactivation by the p65 subunit of NF-kappaB in response to interleukin-1 (IL-1) involves MyD88, IL-1 receptor-associated kinase 1, TRAF-6, and Rac1. Mol Cell Biol.

[25] Wilkinson B., Wang H., Rudd C.E. (2004). Positive and negative adaptors in T-cell signalling. Immunology.

[26] Rudd C.E., Wang H. (2003). Hematopoietic adaptors in T-cell signaling: potential applications to transplantation. Am J Transplant.

[27] Geng L., Rudd C.E. (2002). Signalling scaffolds and adaptors in T-cell immunity. Br J Haematol.

[28] Rudd C.E. (1999). Adaptors and molecular scaffolds in immune cell signaling. Cell.

[29] Medeiros R.B., Burbach B.J., Mueller K.L., Srivastava R., Moon J.J., Highfill S. (2007). Regulation of NF-{kappa}B activation in T cells via association of the adapter proteins ADAP and CARMA1. Science.

[30] Sommer K., Guo B., Pomerantz J.L., Bandaranayake A.D., Moreno-García M.E., Ovechkina Y.L. (2005). Phosphorylation of the CARMA1 linker controls NF-[kappa]B activation. Immunity.

[31] Marinari B., Costanzo A., Viola A., Michel F., Mangino G., Acuto O. (2002). Vav cooperates with CD28 to induce NF-kappaB activation via a pathway involving Rac-1 and mitogen-activated kinase kinase 1. Eur J Immunol.

[32] Turner M., Billadeau D.D. (2002). VAV proteins as signal integrators for multi-subunit immune-recognition receptors. Nat Rev Immunol.

[33] Tybulewicz V.L.J. (2005). Vav-family proteins in T-cell signalling. Curr Opin Immunol.

[34] Raab M., Pfister S., Rudd C.E. (2001). CD28 signaling via VAV/SLP-76 adaptors: regulation of cytokine transcription independent of TCR ligation. Immunity.

[35] Tavano R., Gri G., Molon B., Marinari B., Rudd C.E., Tuosto L. (2004). CD28 and lipid rafts coordinate recruitment of Lck to the immunological synapse of human T lymphocytes. J Immunol.

[36] Dennehy K.M., Kerstan A., Bischof A., Park J.H., Na S.Y., Hunig T. (2003). Mitogenic signals through CD28 activate the protein kinase C theta-NF-kappaB pathway in primary peripheral T cells. Int Immunol.

[37] Schneider H., Cai Y.C., Prasad K.V., Shoelson S.E., Rudd C.E. (1995). T cell antigen CD28 binds to the GRB-2/SOS complex, regulators of p21ras. Eur J Immunol.

[38] Schneider H., Rudd C.E. (2008). CD28 and Grb-2, relative to Gads or Grap, preferentially co-operate with Vav1 in the activation of NFAT/AP-1 transcription. Biochem Biophys Res Commun.

[39] Takeda K., Harada Y., Watanabe R., Inutake Y., Ogawa S., Onuki K. (2008). CD28 stimulation triggers NF-kappaB activation through the CARMA1-PKCtheta-Grb2/Gads axis. Int Immunol.

[40] Marinari B., Costanzo A., Marzano V., Piccolella E., Tuosto L. (2004). CD28 delivers a unique signal leading to the selective recruitment of RelA and p52 NF-kappaB subunits on IL-8 and Bcl-xL gene promoters. Proc Natl Acad Sci U S A.

[41] Piccolella E., Spadaro F., Ramoni C., Marinari B., Costanzo A., Levrero M. (2003). Vav-1 and the IKK alpha subunit of I kappa B kinase functionally associate to induce NF-kappa B activation in response to CD28 engagement. J Immunol.

[42] Cai Y.C., Cefai D., Schneider H., Raab M., Nabavi N., Rudd C.E. (1995). Selective CD28pYMNM mutations implicate phosphatidylinositol 3-kinase in CD86–CD28-mediated costimulation. Immunity.

[43] Riha P., Rudd C.E. (2010). CD28 co-signaling in the adaptive immune response. Self Nonself.

[44] Jain J., Loh C., Rao A. (1995). Transcriptional regulation of the IL-2 gene. Curr Opin Immunol.

[45] Weaver J.R., Good K., Walters R.D., Kugel J.F., Goodrich J.A. (2007). Characterization of the sequence and architectural constraints of the regulatory and core regions of the human interleukin-2 promoter. Mol Immunol.

[46] Kim H.H., Tharayil M., Rudd C.E. (1998). Growth factor receptor-bound protein 2 SH2/SH3 domain binding to CD28 and its role in co-signaling. J Biol Chem.

[47] Medeiros R.B., Burbach B.J., Mueller K.L., Srivastava R., Moon J.J., Highfill S. (2007). Regulation of NF-kappaB activation in T cells via association of the adapter proteins ADAP and CARMA1. Science.

[48] Mueller K.L., Thomas M.S., Burbach B.J., Peterson E.J., Shimizu Y. (2007). Adhesion and degranulation-promoting adapter protein (ADAP) positively regulates T cell sensitivity to antigen and T cell survival. J Immunol.

[49] Burbach B.J., Srivastava R., Medeiros R.B., O’Gorman W.E., Peterson E.J., Shimizu Y. (2008). Distinct regulation of integrin-dependent T cell conjugate formation and NF-kappa B activation by the adapter protein ADAP. J Immunol.

[50] Wang H., Moon E.Y., Azouz A., Wu X., Smith A., Schneider H. (2003). SKAP-55 regulates integrin adhesion and formation of T cell-APC conjugates. Nat Immunol.

[51] Wang H., Rudd C.E. (2008). SKAP-55, SKAP-55-related and ADAP adaptors modulate integrin-mediated immune-cell adhesion. Trends Cell Biol.

[52] Geng L., Pfister S., Kraeft S.K., Rudd C.E. (2001). Adaptor FYB (Fyn-binding protein) regulates integrin-mediated adhesion and mediator release: Differential involvement of the FYB SH3 domain. Proc Natl Acad Sci U S A.

[53] da Silva A.J., Li Z., de Vera C., Canto E., Findell P., Rudd C.E. (1997). Cloning of a novel T-cell protein FYB that binds FYN and SH2-domain-containing leukocyte protein 76 and modulates interleukin 2 production. Proc Natl Acad Sci U S A.

[54] Musci M.A., Hendricks-Taylor L.R., Motto D.G., Paskind M., Kamens J., Turck C.W. (1997). Molecular cloning of SLAP-130, an SLP-76-associated substrate of the T cell antigen receptor-stimulated protein tyrosine kinases. J Biol Chem.

[55] Michel F., Attal-Bonnefoy G., Mangino G., Mise-Omata S., Acuto O. (2001). CD28 as a molecular amplifier extending TCR ligation and signaling capabilities. Immunity.

[56] Harhaj E.W., Maggirwar S.B., Good L., Sun S.C. (1996). CD28 mediates a potent costimulatory signal for rapid degradation of IkappaBbeta which is associated with accelerated activation of various NF-kappaB/Rel heterodimers. Mol Cell Biol.

[57] Kingeter L.M., Paul S., Maynard S.K., Cartwright N.G., Schaefer B.C. (2010). Cutting edge: TCR ligation triggers digital activation of NF-kappaB. J Immunol.

[58] Koretzky G.A., Abtahian F., Silverman M.A. (2006). SLP76 and SLP65: complex regulation of signalling in lymphocytes and beyond. Nat Rev Immunol.

[59] Wang D., Matsumoto R., You Y., Che T., Lin X.Y., Gaffen S.L. (2004). CD3/CD28 costimulation-induced NF-kappaB activation is mediated by recruitment of protein kinase C-theta, Bcl10, and IkappaB kinase beta to the immunological synapse through CARMA1. Mol Cell Biol.

[60] Boomer J.S., Green J.M. (2010). An enigmatic tail of CD28 signaling. Cold Spring Harb Perspect Biol.

